# Dietary aluminium intake disrupts the overall structure of gut microbiota in Wistar rats

**DOI:** 10.1002/fsn3.2955

**Published:** 2022-06-20

**Authors:** Bo Wang, Caihong Wu, Lianzhi Cui, Hui Wang, Ya Liu, Weiwei Cui

**Affiliations:** ^1^ Department of Nutrition and Food Hygiene, School of Public Health Jilin University Changchun China; ^2^ Department of Physical and Chemical Test Jilin Provincial Center for Disease Control and Prevention Changchun China; ^3^ Department of Clinical Laboratory Jilin Cancer Hospital Changchun China

**Keywords:** 16S rRNA sequencing, dietary aluminium, food safety, gut microbiome

## Abstract

Approximately, 40% of ingested dietary aluminium accumulates in the intestine, which has been considered a target organ for dietary aluminium exposure. The gut microbiota may be the first protective barrier against the toxic metal aluminium and a crucial mediator of the bioavailability of metal aluminium. We previously evaluated dietary aluminium intake and its health risks in a population from Jilin Province, China, and found that the average daily intake of aluminium in the diet of residents in Jilin Province was 0.163 mg/kg after the total diet survey. In the present study, the equivalent concentration of aluminium in rats was extrapolated by the average dietary aluminium intake in the population of Jilin Province based on body surface area. Furthermore, healthy adult Wistar rats were randomly divided into four groups (*n* = 15 for each group): a control group and three groups treated with aluminium solution (1, 10, and 100 mg/kg/day, intragastrically) for 28 days. Following treatment, necrosis of renal tubular epithelial cells, hyperplasia of bile ducts and hyperplasia of heart tissue, as well as fiber in the liver, kidney, and heart tissues of aluminium‐treated rats were observed, although there were no significant changes in the spleen and brain. Subsequently, fecal samples were withdrawn for 16S rRNA gene sequence analysis. It was found that aluminium decreased the microbiota diversity and changed the overall community structure of the gut microbiota, including three phyla and four genera, together with the regulation of 12 signaling pathways. Collectively, treatment with aluminium markedly altered the structure of the gut microbiota, suggesting that the disorders of intestinal flora induced by aluminium may be an important mechanism for aluminium toxicity.

## Graphical abstract

Aluminium could decrease the microbiota diversity, change the overall structure of the gut microbiota, including three phyla and four genera, together with regulations of 12 signalling pathways. Treatment with aluminium markedly altered the structure of the gut microbiota, suggesting that the disorders of intestinal flora induced by aluminium may be an important mechanism for aluminum toxicity. It also further reminds people to reduce the use of aluminium‐containing food additives during food processing or replace them with other alternatives as far as possible, and protects people's health by maintaining the homeostasis of intestinal flora through a healthy diet.

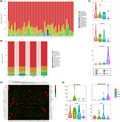

## 1 INTRODUCTION

Aluminium is one of the most abundant metallic elements on earth, and it can be found in soil, water, and air (Stahl et al., 2018). The major source of exposure to aluminium for the general population is through the diet. Aluminium is naturally present in different foods, such as cereals and cereal products (such as pastries, cakes, biscuits, and bread), beverages (such as cocoa and tea), vegetables (such as radishes, lettuce, spinach, mushrooms, and potatoes), and various infant formulae (Yang et al., 2014). In addition, a variety of aluminium compounds have been produced and developed as food and additives and are widely used as curing agents, bulking agents, stabilizers, anticaking agents, and coloring agents in the processing of a wide range of foods such as bread, buns, cakes, muffins, pancakes, and waffles (Aguilar et al., 2008; Aluminium in food, 1994). Furthermore, aluminium foil is mainly used for food packaging and is broadly used in the culinary preparation of various foods, resulting in trace aluminium leaching into food from packaging materials (Takeda et al., 1998). Prolonged consumption of food contaminated with aluminium metal may cause disruption of numerous metabolic processes and subsequently may lead to toxicity to the nervous, skeletal, and hematopoietic systems in addition to impediment in immunological responses, intrauterine growth retardation, and numerous types of cancer (Klotz et al., 2017; Martinez et al., 2018). Aluminium has also been controversially connected with Alzheimer's disease, osteomalacia, and dialysis encephalopathy (Becaria et al., 2002; Kawahara & Kato‐Negishi, 2011). Among the possible toxicity exposures to aluminium, food is an important and unavoidable pathway for human beings. Based on occurrence data for food in combination with consumption data, the survey on aluminium exposure highlighted that 0.2% of adults and 1.6% of children in France (Arnich et al., 2012), almost all the population in China, and a part of the population in Canada and European countries (González‐Weller et al., 2010; Yang et al., 2014) consume more aluminium than the health‐based guidance value of 143 mg/kg body weight/day established by the European Food Safety Authority (EFSA) (Millour et al., 2011), considering food as the sole source of exposure to aluminium. We previously evaluated dietary aluminium intake and its health risks in a population from Jilin Province, China, and found that the highest contributor to aluminium in the diet was from cereals and cereal products; moreover, there may be potential risks to the 2‐ to 6‐year‐old population, as the mean exposure to aluminium for this group was the highest of all (Wang et al., 2020). Thus, increasing dietary contamination with aluminium has become a major global concern of food safety. Although regulatory guidelines for the aluminium contamination level in foods were available, in view of increasing pollution, regular study of contamination in foods is required to prevent the associated health risk.

Aluminium in food or water is mainly absorbed through the gastrointestinal tract, which varies with different aluminium compounds (Hao et al., 2022). Approximately, 40% of ingested aluminium accumulates in the gut, which has been considered a target organ for aluminium exposure (Vignal et al., 2016). Wudi et al. reported that aluminium can promote the apoptosis of intestinal epithelial cells, destroy the structure of tight‐junction proteins, and increase intestinal permeability, injuring the intestinal mucosa barrier (Hao et al., 2022; Lerner, 2012). Several studies have recently identified the gut microbiota as the first protective barrier against toxic metals, including aluminium (Yu et al., 2021). Accordingly, the gut microbiota may be a crucial mediator of the bioavailability of these metals (Breton et al., 2013; Tinkov et al., 2018). For example, the gut microbiota may interact with metals, including aluminium, either through active absorption or passive binding (Breton et al., 2013). Moreover, the gut microbiota and its metabolites, including SCFAs, can influence the transfer of aluminium into the body indirectly, which could affect intestinal barrier integrity (Claus et al., 2011). Meanwhile, toxic metals, including aluminium, may induce changes in the gut microbiota that lead to, or exacerbate, the toxicities associated with these metals (Yu et al., 2021). However, the changes in gut microbial community structure by excessive dietary aluminium intake are currently unclear for humans. In the present study, the equivalent concentration of aluminium in rats was extrapolated by the average dietary aluminium intake in the population of Jilin Province based on body surface area; moreover, healthy rats were administered different doses of aluminium to further investigate the effects of excessive aluminium on gut microbial composition in vivo and clarify the regulation of aluminium metabolism. The highlight of this study lies in the theoretical basis for the participation of gut microbiota in aluminium, which disrupts the intestinal mucosa barrier.

## 2 MATERIALS AND METHODS

### 2.1 Preparation of aluminium solution

We previously evaluated dietary aluminium intake and its health risks in a population from Jilin Province, China, and found that the average daily intake of aluminium in the diet of residents in Jilin Province was 0.163 mg/kg after the total diet survey (Wang et al., 2020). The human dose can be extrapolated to an animal equivalent dose (AED) by a conversion based on body surface area, as previously reported (Nair & Jacob, 2016). AED can be calculated from the following formula:

$$\mathrm{AED}\;\left(\mathrm{mg}/\mathrm{kg}\right)=\text{human dose in}\;\mathrm{mg}/\mathrm{kg}\times \left(\text{human weight in}\;\mathrm{kg}/\text{animal weight in}\;\mathrm{kg}\right).$$

The equivalent concentration of aluminium in rats was calculated by the above equation to be 1 mg/kg. AlCl_3_·6H_2_O was used to prepare the aluminium solution in this experiment. AlCl_3_·6H_2_O (89.5 g) was dissolved in deionized water at 60 °C and then titrated with 0.1 M NaOH solution to pH = 6.5, and distilled water was added to a constant volume of 100 ml to obtain a 10 mg/ml aluminium solution. Then, 1 ml and 10 ml aluminium solutions (10 mg/ml) were taken and diluted to 100 ml with deionized water to obtain 0.1 mg/ml and 1 mg/ml aluminium solutions, which were autoclaved after preparation.

### 2.2 Animals and experimental design

Seven‐week‐old Wistar rats were purchased from Changchun Yisi Experimental Animal Science and Technology Co. Ltd. All animals were housed in specific pathogen‐free cages in the laboratory animal service center at 26 ± 1°C and a humidity of 60 ± 5% under specific pathogen‐free conditions. All animal experiments were approved by the Animal Care Committee of Jilin University. Autoclaved food and water were provided for the mice, and autoclaved bedding was changed twice weekly. Experiments were performed in accordance with published National Institutes of Health guidelines.

After acclimatizing for a week and weighing, rats were randomly assigned into 4 groups (*n* = 15 per group), including a control © group that received a gavage of normal saline and three treatment groups (Al‐L, Al‐M, and Al‐H groups) administered a gavage of aluminium (Al‐L with 1 mg/kg, Al‐M with 10 mg/kg, and Al‐H with 100 mg/kg body weight) once daily for 28 consecutive days. At the same time, the body weight and food intake of all rats were observed and recorded weekly during the experimental period. At the termination of the study, after 12 h of food deprivation, the excrement of rats was collected and frozen in liquid nitrogen. Then, all rats were sacrificed under deep diethyl ether anesthesia, and organ tissues (heart, liver, spleen, brain, and kidney) were excised, weighed, and partially stored in liquid nitrogen immediately.

### 2.3 Determination of viscera index

The viscera index is a biological characteristic index, and the index size can reflect the workload of the organ in the organism to a certain extent (Chen et al., 2021). At the end of the experiment, all rats were sacrificed, tissue samples were collected and weighed, and the viscera indices were calculated with the equation: viscera index = organ weight (mg)/body weight (g) × 100% (Liu et al., 2018).

### 2.4 Histopathology assays

The main organs of all rats were quickly removed, cleaned using cold saline, fixed with 4% buffered formalin, and embedded in paraffin. Next, these fixed organs were stained using hematoxylin and eosin (H&E) to observe histological features. Subsequently, these stained sections were visualized and photographed under an inverted microscope (200× magnification).

### 2.5 16S rRNA gene sequence analysis of the gut microbiota in fecal samples

All rats were fasted for 12 h but were allowed to drink water to avoid the effects of food on the outcome before fecal samples were collected. The obtained fecal samples were stored at −80°C to avoid repeated freeze–thaw cycles. Additionally, fecal samples of rats (*n* > 6) were used for 16S rRNA analysis. Sequencing was performed by Personalbio Co., Ltd., Shanghai, China. A 0.8% agarose gel was used to evaluate the purity and quality of genomic DNA. Total genomic DNA of cecal microbiota was extracted by the QIAamp DNA Stool Mini kit. The total DNA in fecal samples was quantified using a UV spectrophotometer. The V3‐V4 region of the bacterial 16S rRNA gene was amplified by polymerase chain reaction (PCR) using Q5 High‐Fidelity DNA polymerase, and the PCR products were purified using the AP‐GX‐500 DNA Gel Extraction Kit. The DNA library was built up using the Illumina MiSeq platform and sequenced by a MiSeq sequencer.

### 2.6 Bioinformatics analysis

The trimmed and assembled sequences from each sample were aligned to the Greengenes 16S rRNA database using the best‐hit classification option to classify the taxonomy abundance in QIIME. The obtained sequences were merged and divided into operational taxonomic units (OTUs) with 97% sequence similarity using the UCLUST function in QIIME. A Venn diagram was generated to compare OTUs between groups, using QIIME‐calculated Chao1 and Shannon indices for diversity evaluation. Partial least squares discriminant analysis (PLS‐DA) was performed by R software. The co‐occurrence analysis among genera or species was conducted by calculating Spearman's rank correlations of the 50 most abundant genera or species using Mothur software. Network analysis at the genus level with rho >0.06 and *p* < .01 was visualized using Cytoscape. Microbial functions were predicted using PICRUSt. The predicted function spectrum data were clustered according to the abundance distribution of functional groups or the similarity between samples and presented by a heatmap; the predicted genes and their functions were aligned to the Kyoto Encyclopedia of Genes and Genomes (KEGG) database.

### 2.7 Statistical analysis

The results were presented as the mean ± standard deviation (SD). Differences were evaluated by one‐way analysis of variance (ANOVA) followed by Tukey's post hoc test using IBM SPSS Statistics 26.0. *p* values <.05 were significant; *p < .05, **p < .01.

## 3 RESULTS

### 3.1 Effects of aluminium on body weight, food intake, and major organs in rats

**FIGURE 1:**
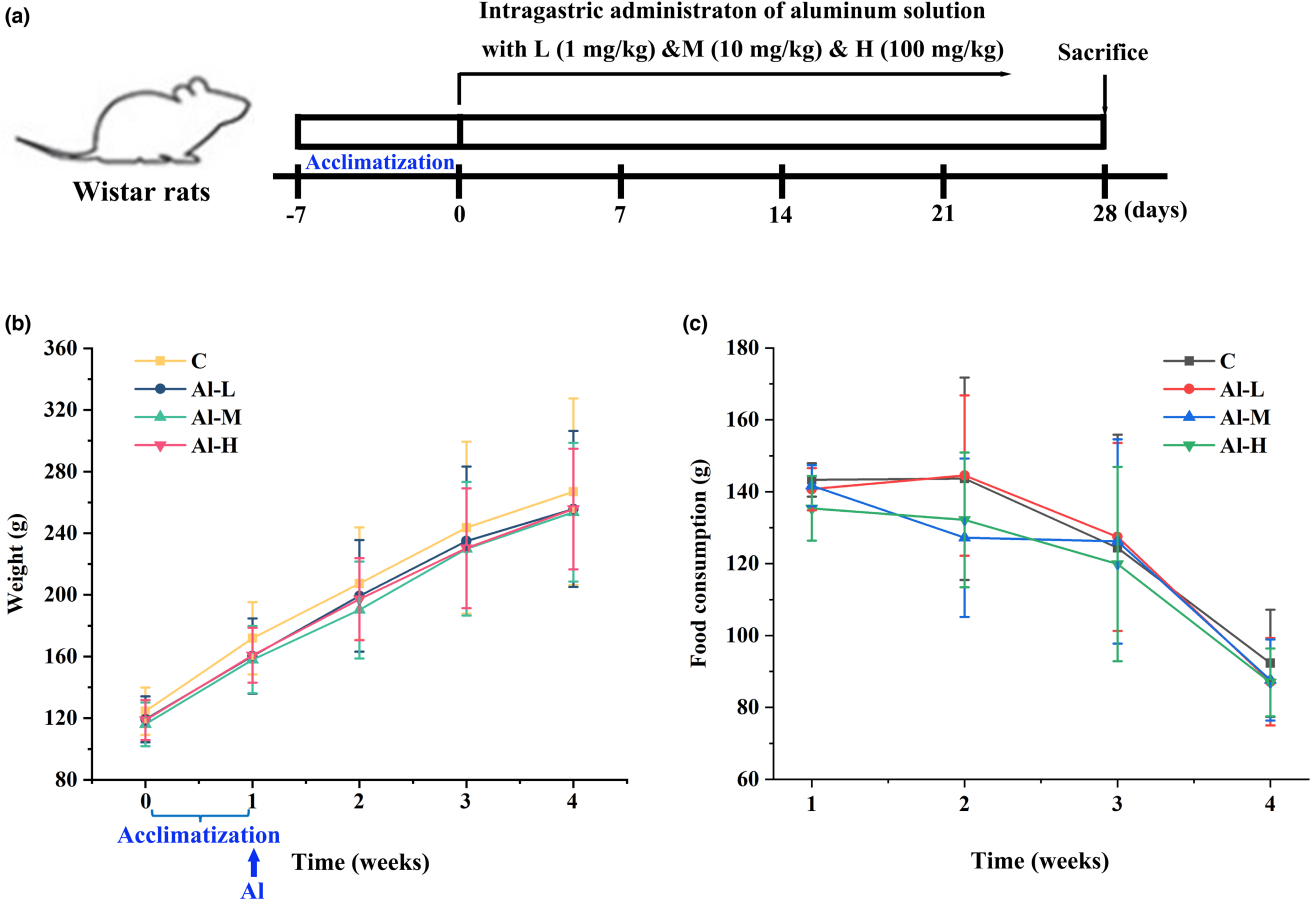
Effects of aluminium on body weight and food consumption of rats. (a) Schematic overview of the experimental design. (b, c) Changes in body weight and food consumption of different groups are shown for Wistar rats administered aluminium solution for 4 weeks. Data are represented as the means ± standard deviation (SD) (*n* > 6)

**FIGURE 2:**
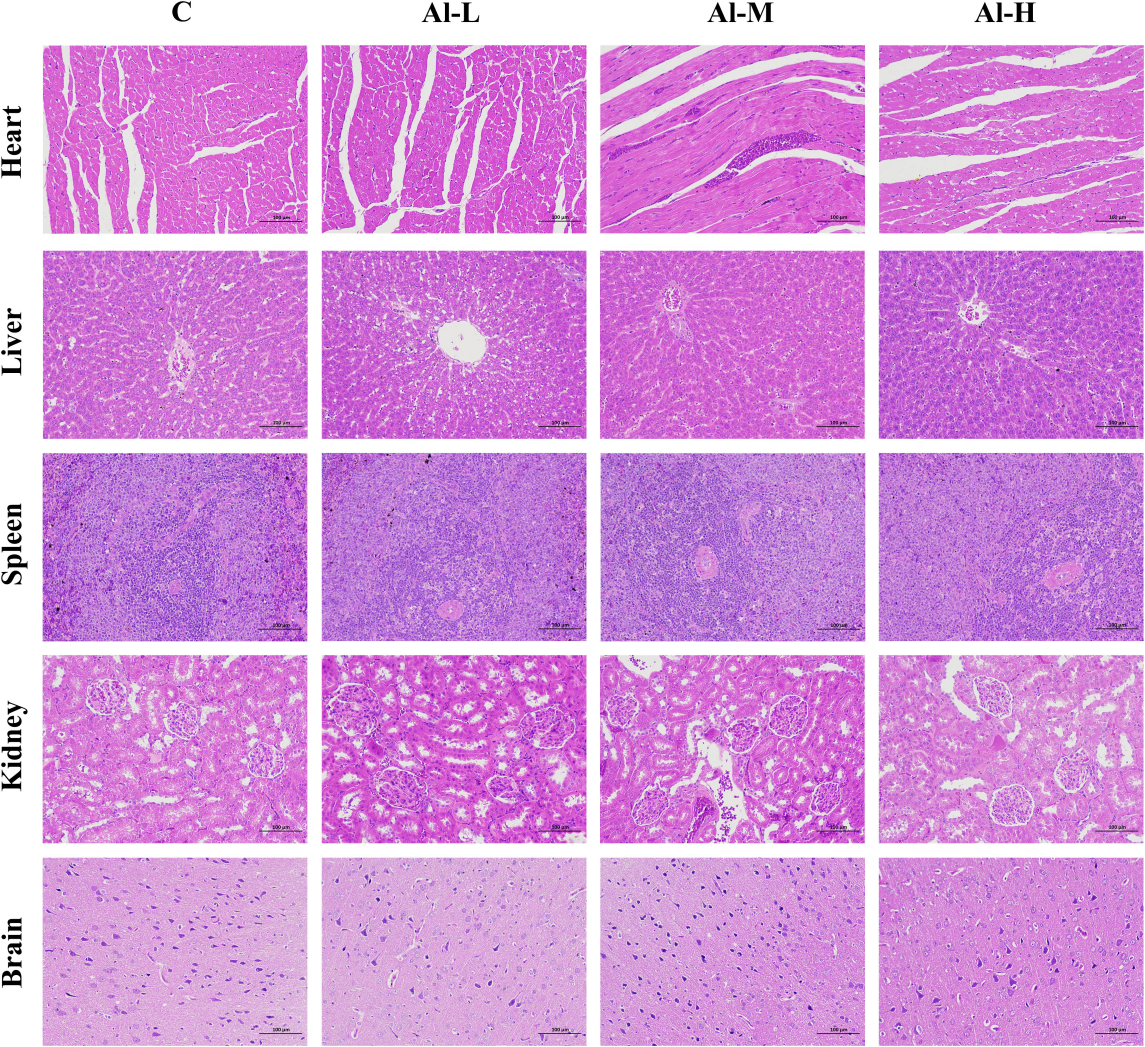
Representative H&E‐stained slices of major organs (heart, liver, spleen, kidney, and brain)

To investigate the effect of aluminium on living organisms, healthy rats were intragastrically administered aluminium solution (Al‐L with 1 mg/kg, Al‐M with 10 mg/kg, and Al‐H with 100 mg/kg) every day for 28 consecutive days (Figure 1a). The body weight and food consumption of all rats from different groups were monitored weekly. As shown in Figure 1b, the food intake of rats gradually decreased with the extension of feeding time. The body weight of the aluminium‐treated groups decreased compared with that of the control group, but there was no statistically significant difference. In addition, food consumption and viscera index of rats for all the groups did not differ significantly (Figure 1c and Table 1). Furthermore, based on H&E staining, a normal histopathological structure of the myocardium was observed in the hearts of control rats, and the aluminium‐treated groups showed focal desquamation of endocardial lining cells with inflammatory cell infiltration and hemorrhage in the underlying myocardium. We observed centrilobular congestion and congestion of sinusoids with neutrophilic infiltration in aluminium‐treated rats compared with the control group. Kidney sections of control rats showing glomeruli and tubules appeared normal, and tubular cell swelling, nuclear condensation, and tubular dilatation were observed in aluminium‐treated rats. However, aluminium treatment had no significant changes in the spleen and brain of rats (Figure 2). These results suggest that aluminium causes significant damage to major organs (heart, liver, and kidney).

**TABLE 1:** Visceral index

Group	Heart	Liver	Spleen	Brain	Kidney
C	3.85 ± 1.01	41.92 ± 13.47	3.44 ± 1.17	6.09 ± 0.78	9.44 ± 2.32
Al‐L	3.60 ± 1.01	35.84 ± 8.07	2.75 ± 0.74	6.46 ± 0.35	8.00 ± 1.56
Al‐M	3.73 ± 1.01	41.86 ± 11.44	3.02 ± 0.57	6.06 ± 0.67	9.10 ± 1.48
Al‐H	3.60 ± 1.01	40.72 ± 5.62	2.99 ± 0.67	6.19 ± 0.80	8.96 ± 0.97

### 3.2 Aluminium alters the overall structure of the gut microbiome in rats

**FIGURE 3:**
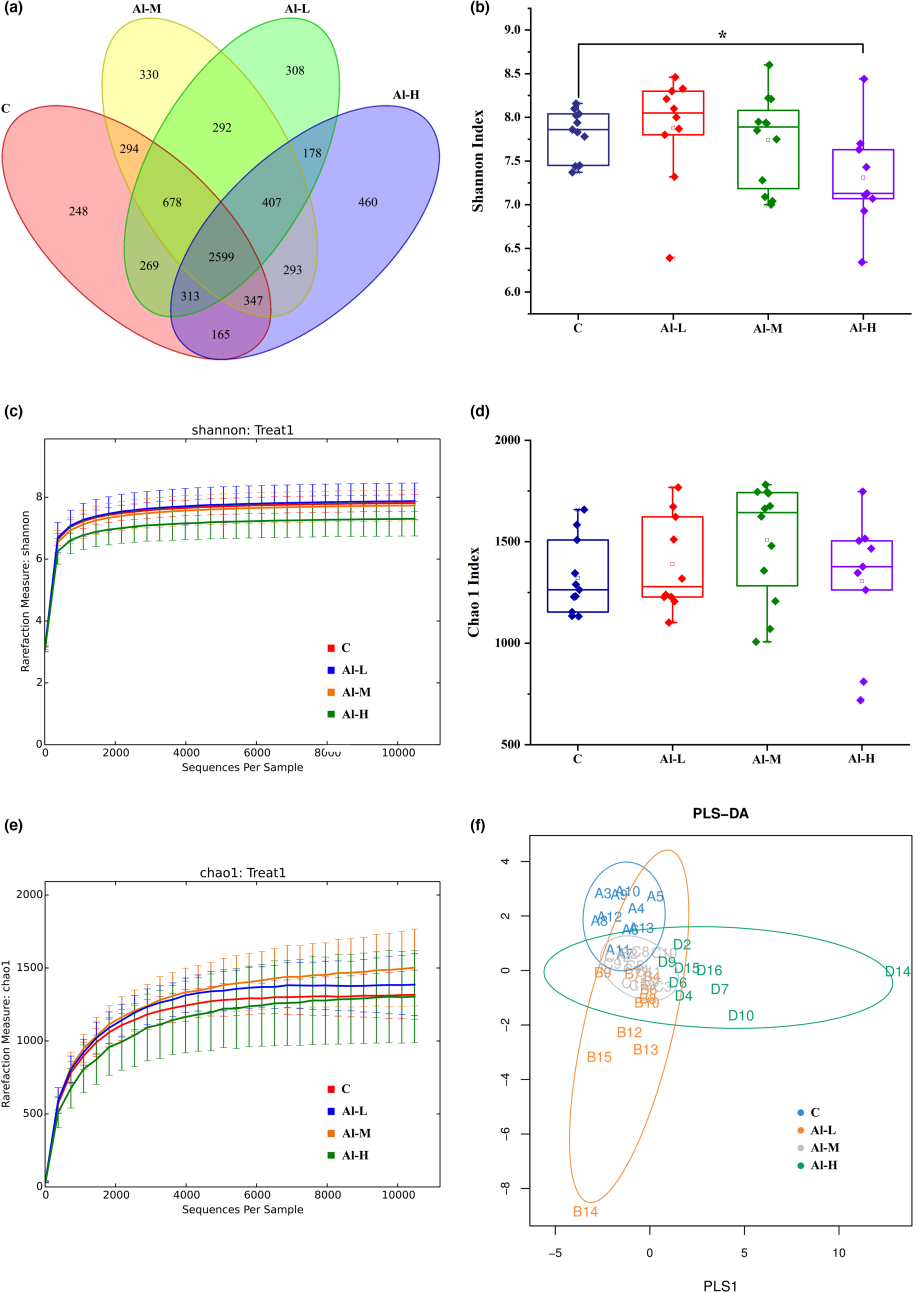
Diversity analysis of the fecal microbiota communities based on OTUs. (a) Venn diagram of different groups. (b, d) Comparison of the richness and diversity indices of different groups. (c) Chao1 rarefaction curve. (e) Shannon rarefaction curve. (f) Evaluation of the community structure of intestinal microbes from 16S rRNA sequencing using PLS‐DA in different groups. Data are represented as the means ± SD (*n* > 6); *p < .05

To determine the changes in the gut microbial community triggered by aluminium treatment, high‐throughput sequencing of 16S rRNA in the fecal samples from rats was performed by the Illumina MiSeq sequencing system. Altogether 3,469,001 sequences were obtained from all the fecal samples, averaging 34,690 sequences in each sample (27,636–61,785). All sample sequences were converted into operational taxonomic units (OTUs). The abundance of OTUs was counted to make the Venn diagram. As shown in Figure 3a, there were 248 unique OTUs in the C group, 308 in the Al‐L group, 330 in the Al‐M group, and 460 in the Al‐H group, while 2599 common OTUs existed in all groups. Compared to that of the control group, the average number of OTUs in the aluminium‐treated groups was significantly increased.

The intestinal microbiota richness and diversity of different treatment groups were assessed using Chao 1 and Shannon indices, respectively. The Chao1 index was used to evaluate the community richness. The characterization of community diversity and uniformity was assessed by the Shannon index. As shown in Figure 3d, we found that aluminium treatment caused a decrease in the diversity of the gut microbiome as that of the control group, but only to a significant degree in the high‐dose aluminium solution‐treated rats (Shannon index, *p* < .05), although no significant differences in microbial richness were observed between the aluminium‐treated groups and the control group (Figure 3b). In addition, the species rarefaction curve (Figure 3c,e) also presented the same tendency in the richness (Chao1 index) and diversity (Shannon index) between the C and other groups; moreover, it demonstrated that the current sequencing depth is sufficient to reflect the microbial diversity of the samples. A clear distinction was also observed between the microbial communities of the control group and treated groups, as shown in the PLS‐DA plot. The samples of each treatment group were distinctly clustered, while the aluminium group was isolated from that of the remaining three groups (Figure 3f). The above data indicate that the community structure of the gut microbiome is altered by aluminium treatment.

### 3.3 Aluminium changes the abundance of certain gut microbes

**FIGURE 4:**
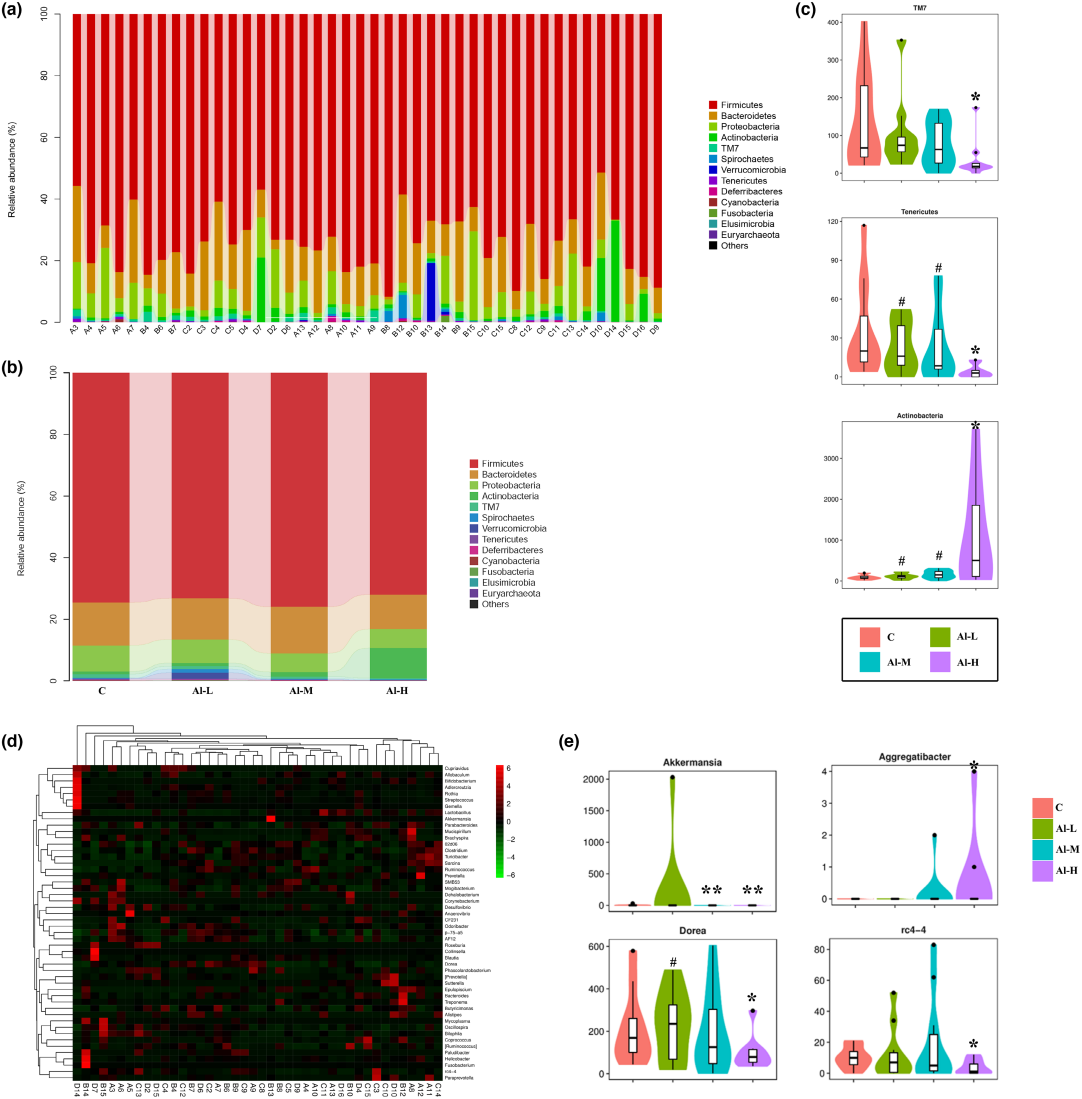
Taxonomy analysis of microbiota components. (a) Relative abundance of the phylum from each sample. (b) The average abundance of each phylum in the control group, Al‐L group, Al‐M group, and Al‐H group. (c) Significant intergroup differences were found in the three phyla. (d) Heatmap showing the relative abundance of genera ranking in the top 50 from each sample. (e) Significant intergroup differences were found in four genera. Data are represented as the means ± SD (*n* > 6); *p < .05, **p < .01 versus the C group; ^**#**^ *p* < .05 versus the Al‐H group

To identify the intestinal differential microbes altered by aluminium treatment, we examined changes in bacterial abundance at the phylum and genus levels by taxon analysis. A total of 13 phyla were identified by all samples at the phylum level (Figure 4a,b). High‐dose aluminium treatment significantly reduced the relative abundance of Tenericutes and TM7 (*p* < .05), but increased the Actinobacteria level (*p* < .05). Meanwhile, compared to the Al‐H group, the abundance of Tenericutes and Actinobacteria exhibited significant differences in the Al‐L and Al‐M groups (*p* < .05) (Figure 4c). At the genus level, as shown in Figure 4d,e, aluminium significantly increased the abundance of Aggregatibacter (*p* < .05), while it lowered the abundance of three genera, including Akkermansia (*p* < .01), Dorea (*p* < .05) and rc4‐4 (*p* < .05). The above data show that aluminium changes the overall structure of the gut microbiome by controlling the abundance of certain bacteria.

### 3.4 The function of the gut microbiota changed after aluminium treatment

**FIGURE 5:**
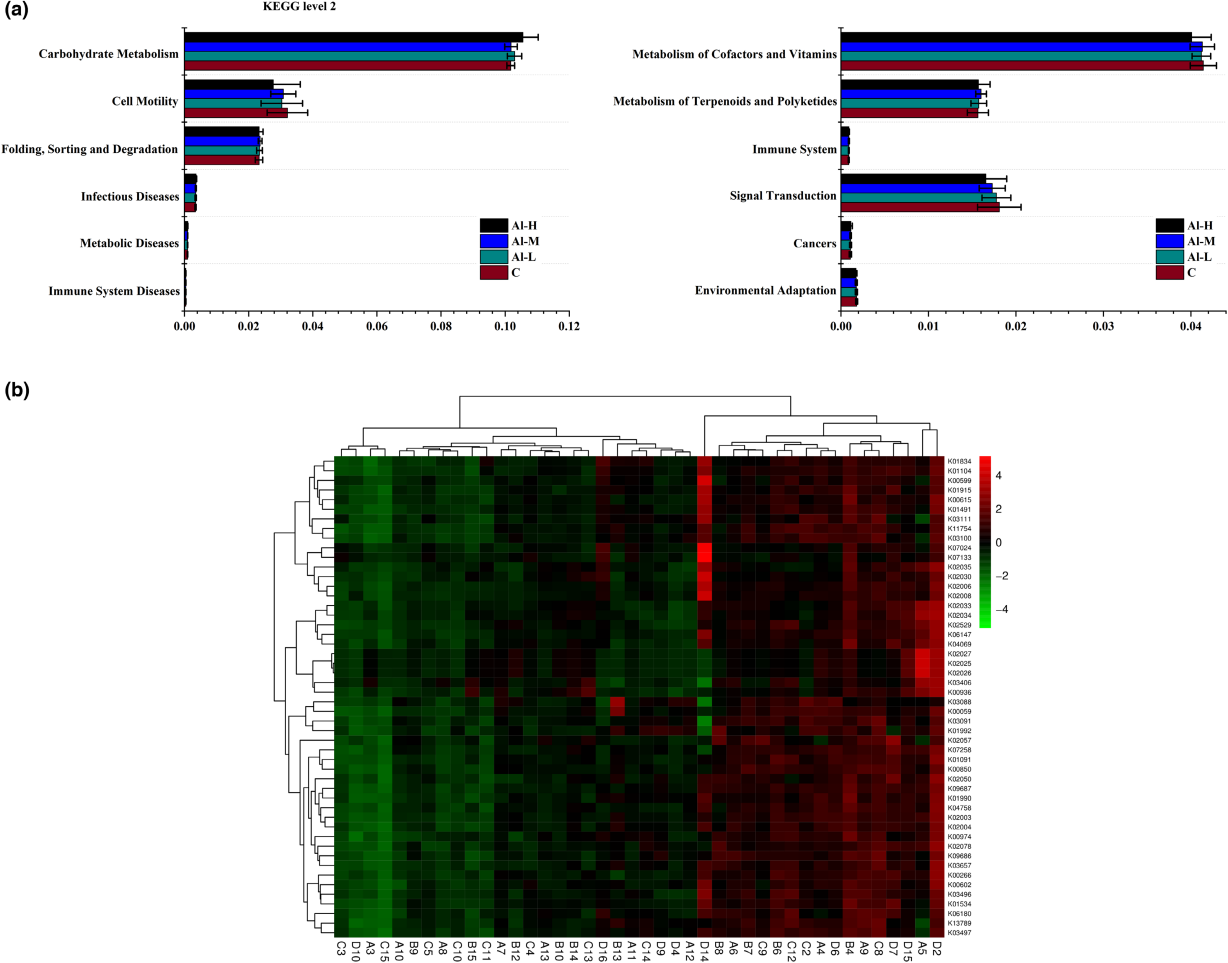
Microbiome function prediction according to the Kyoto Encyclopedia of Genes and Genomes (KEGG) pathway database. (a) KEGG level 2 metabolic pathways. (b) Heatmap showing the specific annotated information of each KEGG orthologous group (KO) in the metabolic pathway

To understand whether functional genes change with the structure of the gut microbiota, we employed PICRUSt to predict the microbial functions of the members among different groups. The 16S rRNA gene sequence of the microbiota was calculated in the KEGG database, and the average abundance of the metabolic pathway was obtained according to the database. We found that 12 functions were represented overall (Figure 5a). Among them, the trend of 7 metabolism pathways was upregulated in aluminium‐treated rats compared with the control group, including carbohydrate metabolism, infectious diseases, metabolic diseases, metabolism of terpenoids and polyketides, immune system, cancers, and folding, sorting and degradation. However, the pathways involved in the metabolism of cofactors and vitamins, immune system diseases, signal transduction, cell motility, and environmental adaptation pathways were downregulated. Cluster analysis of the heatmap showed that the functional spectrum of the aluminium‐treated group was altered compared to that of the control group (Figure 5b). These data suggest that the changes in microbial function are caused by aluminium‐induced gut microbiota disorders in Wistar rats.

## 4 DISCUSSION

With the rapid development of the social economy, increasing dietary contamination with aluminium has become a serious global concern for food safety. Aluminium occurs naturally in foodstuffs and drinking water and is also widely used in the diet as a food additive, but excessive intake of it in living organisms can be harmful to the body (Jaishankar et al., 2014). Aluminium, a neurotoxicant that affects diverse metabolic reactions, can enter and accumulate in the brain from the systemic circulation or the site of absorption (Igbokwe et al., 2019), has been proven to be involved in aluminium‐mediated neurodegeneration resulting in cognitive dysfunction, and may cause Alzheimer's disease (Yokel et al., 2002). Additionally, aluminium has been suspected as an adjuvant for the induction of Crohn's disease (Lerner, 2012), a highly debilitating disease characterized by excessive uncontrolled intestinal inflammation (Ballester Ferré et al., 2018). Recently, it has been demonstrated that ingestion of excessive aluminium worsened intestinal inflammation in mice with chemical induction (Djouina et al., 2016). In addition, aluminium poisoning may generate aluminium‐induced bone disease, brain cancer, and lung cancer (Wasana et al., 2015). Moreover, the human immune system is also sensitive to aluminium exposure (Gräske et al., 2000). An increasing body of evidence implicates aluminium as a potentially hazardous agent. Faced with dietary aluminium intake, the intestinal tract is an essential barrier, especially as 40% of ingested aluminium accumulates at the intestinal mucosa (Vignal et al., 2016). Importantly, the gut microbiota plays vital roles in human physiology and metabolism, and aluminium‐induced changes in these bacteria may be an important mechanism for aluminium toxicity (Yu et al., 2016). There have been few studies on the effects of dietary aluminium on the human gut microbiota; thus, it is necessary to explore the effects of dietary aluminium on the diversity and overall structure of the gut microbiota in humans. We previously evaluated dietary aluminium intake and its health risks in a population from Jilin Province, China, and found that the average daily intake of aluminium in the diet of residents in Jilin Province was 0.163 mg/kg after the total diet survey (Wang et al., 2020). The equivalent concentration of aluminium in rats was extrapolated by the average dietary aluminium intake in the population of Jilin Province based on body surface area (Nair & Jacob, 2016). In the present study, healthy rats were intragastrically administered aluminium solution at different concentrations. We found that the body weights of aluminium‐treated rats were decreased compared to the control group, but there was no statistically significant difference. Moreover, aluminium had no effect on the food consumption and organ index of the rats (Figure 1 and Table 1). Aluminium treatment resulted in necrosis of renal tubular epithelial cells, hyperplasia of bile ducts, and hyperplasia of heart tissue fiber in liver, kidney, and heart tissues, although there were no significant changes in the spleen and brain of rats (Figure 2). Subsequently, 16S rRNA gene sequence analysis of the gut microbiota was performed in fecal samples. As demonstrated by our data, aluminium treatment decreased the diversity of microbiota components and changed the overall microbiome structure compared with normal rats (Figure 3). Concretely speaking, the microbiota community shift was caused by aluminium, of which three phyla and four genera were significantly affected (Figure 4). At the genus level, Aggregatibacter was markedly enriched by aluminium treatment. Aggregatibacter, a facultative gram‐negative bacterium, is a dominant etiology of infective endocarditis caused by fastidious organisms (Nørskov‐Lauritsen, 2014). In addition to endocarditis, Aggregatibacter is a cause of periodontal infection, and, in conjunction with *Actinomyces israelii*, of soft tissue abscesses and pneumonia (Homsi & Kapila, 2020). The remaining three genera, Akkermansia, Dorea, and rc4‐4, were significantly decreased with aluminium treatment. Akkermansia is a mucin‐degrading bacterium (Derrien et al., 2004), accounts for 1 to 4% of the adult intestinal microbiome, and is a species of bacteria that inhabits the large intestine (Belzer & de Vos, 2012; Naito et al., 2018). Akkermansia, as a by‐product of degrading mucin, produces acetate and propionate, two short‐chain fatty acids that serve as nutrients for other beneficial intestinal bacteria that release butyrate, a short‐chain fatty acid and vital energy source for mucus‐secreting goblet cells and intestinal epithelial cells (Zhou, 2017). At the same time, its short‐chain fatty acid metabolites feed intestinal cells and thereby strengthen the intestinal barrier to prevent harmful substances from passing through, ultimately leading to the reduction of inflammation and production of anti‐inflammatory molecules (Zhou, 2017). Akkermansia also helps metabolize fiber, supporting butyrate‐producing bacteria within the gut (Zhou, 2017). Additionally, Plovier et al. demonstrated the effectiveness of Akkermansia at lowering blood lipid levels, insulin resistance, adipose tissue inflammation, and weight loss in mice (Ou et al., 2020; Plovier et al., 2017). Rc4‐4 is considered to be a bacterium associated with diet‐induced obesity (Moorthy et al., 2021). Eunhee et al. have found increased abundance of the rc4‐4 genus with improved metabolic homeostasis, although the genus rc4‐4 has been associated with diet‐induced obesity (Chung et al., 2020). *Dorea* is a genus of obligate‐anaerobic gram‐positive nonspore‐forming bacteria that belongs to the Lachnospiraceae family (Xi et al., 2019). *Dorea* can ferment glucose and other sugars, and the main metabolic products are formic and acetic acids, ethanol, carbon dioxide, and hydrogen, which are provided to living organisms (Xi et al., 2019). All the above findings suggest that aluminium alters the overall structure of the gut microbiome by controlling the abundance of these bacteria.

Recently, it has been suggested that alterations in gut microbiota can lead to changes in cellular components or metabolites in bacterial cells (Vicentini et al., 2021). Predictive functional analyses of sequencing data suggested that aluminium changed various pathways, including metabolites (carbohydrate metabolism, metabolism of terpenoids, and polyketides), genetic information processing (folding, sorting, and degradation), human diseases (immune system diseases, infectious diseases, metabolic diseases, and cancers), and organismal systems (immune system) in control mice (Figure 5). Hence, it is possible that the changes in microbial function are caused by aluminium‐induced gut microbiota disorders in Wistar rats.

## 5 CONCLUSION

In summary, by increasing the necrosis of renal tubule epithelial cells, hyperplasia of bile ducts, and hyperplasia of heart tissue fibers, aluminium caused damage to the main organ tissues (heart, liver, and kidney) of rats, in which the gut microbiome plays a key role. Treatment with aluminium decreases the microbiota diversity, changes the overall structure of the microbiota, alters the growth of three phyla and four genera, and is accompanied by regulations of the regulation 12 signaling pathways. This implies that aluminium can cause changes in microbial function by inducing disorders of the intestinal flora including the function and diversity of the gut microbial community structure.

## CONFLICT OF INTEREST

The authors declare no conflict of interest.

## Data Availability

This is an open access article under the terms of the Creative Commons Attribution License, which permits use, distribution and reproduction in any medium, provided the original work is properly cited.
